# A Simple Index for Nonalcoholic Steatohepatitis—HUFA—Based on Routinely Performed Blood Tests

**DOI:** 10.3390/medicina55060243

**Published:** 2019-06-03

**Authors:** Milica Culafic, Sandra Vezmar Kovacevic, Violeta Dopsaj, Milos Stulic, Zeljko Vlaisavljevic, Branislava Miljkovic, Djordje Culafic

**Affiliations:** 1 Department of Pharmacokinetics and Clinical Pharmacy, Faculty of Pharmacy, University of Belgrade, 11000 Belgrade, Serbia; svezmar@pharmacy.bg.ac.rs (S.V.K.); milbran@pharmacy.bg.ac.rs (B.M.); 2 Clinic for Gastroenterology and Hepatology, Clinical Centre of Serbia, School of Medicine, University of Belgrade, 11000 Belgrade, Serbia; drstulic@gmail.com (M.S.); kcszeljko@gmail.com (Z.V.); djordjeculafic@sbb.rs (D.C.); 3 Department of Medical Biochemistry, Clinical Centre of Serbia, Faculty of Pharmacy, University of Belgrade, 11000 Belgrade, Serbia; violeta.dopsaj@gmail.com

**Keywords:** fatty liver disease, identification, index, NAFLD, NASH

## Abstract

*Background and objectives:* Data suggests that nearly 30% of the general population have steatosis and up to 5% of this population develops nonalcoholic steatohepatitis (NASH). Liver biopsy is still considered to be the gold standard for the diagnosis of NASH. Great effort is being made toward the identification of sensitive diagnostic tests that do not involve invasive procedures to address a common concern in patients with the nonalcoholic fatty liver disease—whether they have NASH or simple steatosis. We aimed to investigate the independent predictors and develop a non-invasive, easy-to-perform, low-cost set of parameters that may be used in clinical practice to differentiate simple steatosis from NASH. Methods: А cross-sectional study of nonalcoholic fatty liver disease (NAFLD) patients divided into two groups: group I—simple steatosis (SS) and group II—biopsy-proven NASH. Strict inclusion criteria and stepwise analysis allowed the evaluation of a vast number of measured/estimated parameters. *Results:* One hundred and eleven patients were included—82 with simple steatosis and 29 with biopsy-proven NASH. The probability of NASH was the highest when homeostatic model assessment of insulin resistance (HOMA-IR) was above 2.5, uric acid above 380 µmol/L, ferritin above 100 µg/L and ALT above 45 U/L. An acronym of using first letters was created and named the HUFA index. This combined model resulted in an area under the receiver operator characteristic curve (AUROC) of 0.94, provided sensitivity, specificity, positive predictive value and a negative predictive value for NASH of 70.3%, 95.1%, 83.1% and 90.0%, respectively. *Conclusion*: We suggest a simple non-invasive predictive index HUFA that encompasses four easily available parameters (HOMA-IR, uric acid, ferritin and ALT) to identify patients with NASH, which may reduce the need for a liver biopsy on a routine basis in patients with NAFLD.

## 1. Introduction

Nonalcoholic fatty liver disease (NAFLD) refers to a group of conditions characterized by fatty deposits in the liver in the absence of alcohol consumption. Excessive accumulation of hepatic fat occurs in a disease spectrum from simple steatosis, through to nonalcoholic steatohepatitis (NASH), fibrosis and cirrhosis, liver failure and hepatocellular carcinoma (HCC). Available data suggest that nearly 30% of the general population have steatosis and up to 5% of this population develops NASH [1]. Early stage NASH patients will progress to cirrhosis in 9% to 20% over a period of 5–10 years [2]. In a systematic review of patients with NAFLD, the risk of HCC among the cirrhotic group ranged from 2.4% over seven years to 12.8% over three years [3]. Higher overall mortality rates in patients with NAFLD compared with the general population have also been reported [4]. In addition, NAFLD is identified to displace viral hepatitis as the primary cause of end-stage liver disease and liver transplantation over the following decade [5].

Confirming accurate staging of liver disease is essential for allowing both a timely therapeutic approach and prognostic evaluations. The diagnosis of NASH, evaluation and staging of fibrosis are based on histological examination of a tissue specimen obtained by liver biopsy. Although this approach is still considered to be the gold standard in all currently available guidelines [6,7,8], liver biopsy cannot be recommended for all patients, considering the limitations (invasiveness, sampling variability and cost) and high prevalence of NAFLD worldwide [9]. There is a risk of complications arising from liver biopsy, from mild ones such as pneumothorax to severe hemorrhage, liver rupture and injury to the biliary system [10]. Attributable to the risk of complications, some patients may refuse a liver biopsy [11]. Moreover, it is less likely that there will be enough medical workforce to perform a liver biopsy on all potential NAFLD patients [12]. The newly published study also reported only 24% of physicians routinely perform a liver biopsy to diagnose NASH [13]. A liver ultrasound (US) examination is a primary imaging method useful for confirming steatosis [14]. Despite the benefits of a US, regarded as being non-invasive, widely available and low cost, it has certain limitations, as well as limited accuracy in obese patients [14,15]. Alternative methods to a liver biopsy in NASH patients are computed tomography (CT), magnetic resonance imaging (MRI) with chemical shift imaging and particularly magnetic resonance spectroscopy and elastography [16,17]. Although imaging methods such as CT and MRI are more sensitive modalities for quantifying steatosis, neither can evaluate liver fibrosis and NASH [18,19]. Non-invasive evaluation of liver fibrosis and NASH may be performed by ultrasound elastography and magnetic resonance elastography. However, none of these imaging techniques has sufficient sensitivity and specificity for staging the disease and cannot distinguish between simple steatosis and NASH [17].

Another non-invasive approach is to develop clinical and biochemical parameters that can identify fibrosis in a cohort of patients with NAFLD, to replace liver biopsy, at least to some extent. Various non-invasive panels have been suggested to stage liver disease [20]. Many currently available tests have certain limitations, which has significant clinical implications [21]. These considerations accentuate the need for non-invasive methods that are inexpensive, reliable and reproducible to distinguish between simple steatosis and NASH. Furthermore, the non-invasive approach may limit the need for a liver rebiopsy and consequently lead to a more efficient follow-up [22,23].

This study aimed to investigate the independent predictors and develop a non-invasive, easy-to-perform, low-cost set of parameters that may be used in clinical practice to differentiate simple steatosis from NASH.

## 2. Materials and Methods

We conducted a cross-sectional study of 111 patients with NAFLD, recruited on an outpatient basis. The study was performed from October 2014 to October 2017. A patient’s eligibility was based on their medical history, physical examination, biochemical testing and liver ultrasound imaging, performed during the screening visit. The inclusion criteria were age >18 years, bright liver on ultrasound imaging and the patient’s consent to participate in the study. The following exclusion criteria were applied: alcohol consumption >20 g/day, other liver diseases (liver cirrhosis, viral hepatitis, autoimmune hepatitis, primary sclerosing cholangitis, primary biliary cirrhosis and overlap syndromes, hemochromatosis, Wilson’s disease, α1-antitrypsin deficiency and medication-induced liver disease), diabetes mellitus type I and II, hypothyroidism or hyperthyroidism, adrenal insufficiency, renal failure, malignant diseases, addiction to any medication and the use of the following medications within a year prior to screening: estrogens, progestins, glucocorticosteroids, thiazolidinediones, orlistat, spironolactone, vitamins E, C and B, folate and iron supplements, ursodeoxycholic acid, tamoxifen, amiodarone, biologic agents and statins.

### 2.1. Patients

Patients were divided into two groups: group I—simple steatosis (SS) and group II—NASH. More specifically, 82 patients were determined to have simple steatosis (out of which 33 had undergone a liver biopsy) while 29 patients had biopsy-proven NASH. Liver biopsies were scored using a NAFLD activity score (NAS) and fibrosis staging according to Kleiner et al. [24].

The histopathologist who reported the liver biopsies was blinded to other parameters. Information on socio-demography, economic and lifestyle characteristics and pre-existing medical conditions were gathered as part of a standardized personal interview. Participants were asked to report their age, smoking status, frequency of alcohol intake, nutritional habits, education and occupation. Body mass index was calculated as body weight (kg) divided by the square of height (m^2^). Waist and hip circumference were measured (in cm) with an inelastic tape in line with WHO guidelines [25]. A blood pressure measurement was performed with patients in the seated position, according to standard protocol. Venous blood was collected from each patient after an overnight fast, serum was used for the biochemical analysis. Insulin resistance was evaluated through the program Homeostasis Model Assessment (HOMA) Calculator v2.2.2 [26] based on the following equation: Fasting insulin (microU/L) × fasting glucose (nmol/L)/22.5.

### 2.2. Laboratory Analyses

Laboratory analyses included the full blood count, erythrocyte sedimentation rate (ESR), C-reactive protein (CRP), standard liver enzymes: Alanine aminotransferase (ALT), aspartate aminotransferase (AST) and gamma-glutamyl transferase (GGT); total cholesterol (TC), high-density lipoprotein (HDL), low-density lipoprotein (LDL), triglycerides (TG), bilirubin, total proteins, albumin, urea, creatinine, iron, ferritin, folate, vitamin B12, insulin and serum uric acid (SUA). All blood samples were aliquoted at the same day and stored at −80 °C for further analysis of leptin, interleukin 6 (IL-6), homocysteine and sulfhydryl groups. These parameters were measured using validated standard methods and devices available at the Medical Biochemistry Department.

### 2.3. Statistical Analysis

Categorical variables are presented as numbers/percentages while continuous variables are shown as mean ± standard deviation, or median with interquartile range Kolmogorov–Smirnov test was used to evaluate the normality of distribution. The statistical analysis was performed in three steps. The first step comprised a simple comparison between groups (SS and NASH) using a chi-square test for categorical variables and *t*-test or Mann–Whitney test for continuous variables. The second step included an analysis of covariance (ANCOVA) to investigate if parameters differ between the observed groups. Spearman’s rank correlation coefficient was applied to correlate the NAS score and fibrosis with the proposed parameters of the HUFA index. The third step included a multivariate logistic regression analysis of the significant independent variables identified in the previous testing. All reported *p*-values were two-tailed, and those less than 0.05 were deemed statistically significant.

The area under the curve of the receiving operating characteristic (ROC) was used to evaluate the sensitivity, specificity, positive predictive value (PPV) and negative predictive value (NPV) of the proposed model for the prediction of NASH. Statistical analysis was performed with the Statistical Package for Social Science version 17.0 software (SPSS, Chicago, IL, USA).

### 2.4. Ethical Considerations

The study was conducted in accordance with Guidelines for Good Clinical Practice, the Declaration of Helsinki and local laws and regulations. The protocol was approved by joint Research and Ethics Committee of the Clinical Centre of Serbia, Belgrade, filed under number 262/2. Written informed consent was obtained from all the participants in the study.

## 3. Results

To identify the predictive factors linked to NASH, we compared variables between groups. Anthropometric and clinical characteristics of the patients included in the study are given in Table 1. No significant differences between groups in age, body mass index (BMI), waist and hip circumferences were detected.

Comparative data of groups, in regards to parameters measured in serum and estimated ones are shown in Table 2. We identified significantly higher values of ALT and AST in conjunction with different AST/ALT ratio as anticipated among the observed groups. Interestingly, we noticed that total proteins, albumin and sulfhydryl groups, total cholesterol, LDL, SUA ferritin, insulin and HOMA index were also significantly different between NASH and SS groups.

Parameters that have shown statistical significance in observed patient groups SS vs. NASH (*p* < 0.05) were further entered into the ANCOVA analysis to control for confounding variables (age, sex and BMI). The results showed that sulfhydryl groups, CRP, ESR, ferritin and SUA were statistically different between SS and NASH groups at the level *p* < 0.05, while the statistical significance between these groups were more pronounced (*p* < 0.001) for ALT, AST/ALT ratio and homeostatic model assessment of insulin resistance (HOMA-IR). Furthermore, sulfhydryl groups, CRP, ESR and the AST/ALT ratio yielded an area under the receiver operator characteristic curve (AUROC) of 0.642, 0.332, 0.277 and 0.524, respectively. AUROC less than 0.7 is considered a low-performance indicator, thus those parameters were omitted from the final model. Lastly, the final model was created based on the four remaining variables. 

Histological characteristics of NASH patients based on the NAS score were compared to selected variables from the final model (Table 3).

**Figure 1:**
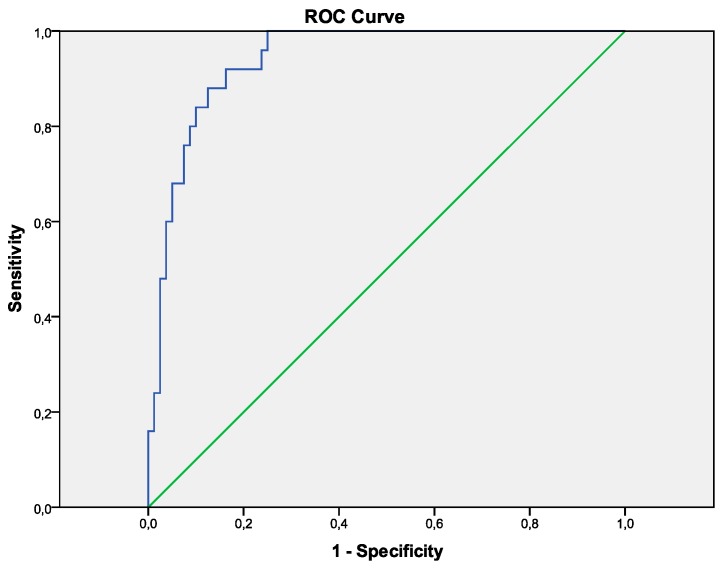
Curve for the prediction of NASH by the HUFA index. Calculator based on the suggested model is available upon request.

The probability of NASH was the highest when HOMA-IR was above 2.5, uric acid above 380 µmol/L, ferritin above 100 µg/L and ALT above 45 U/L. This combined model resulted in a higher area under the ROC curve (0.94)than for any individual model, provided sensitivity, specificity, positive predictive value and negative predictive value for NASH of 70.3%, 95.1%, 83.1% and 90.0%, respectively (Table 4 and Table 5, Figure 1).

## 4. Discussion

Screening for significant liver injury in patients with NAFLD will develop into a critical medical challenge in the near future, owing to the epidemic proportions of obesity and diabetes. The prevalence of obesity has more than doubled worldwide since 1980, as indicated by the World Health Organization. In 2014 more than 1.9 billion adults were overweight, of whom more than 600 million were obese. The prevalence of simple steatosis in obese patients is 60%, while 20–25% progress to NASH and 2–3% develop cirrhosis [27,28]. It is estimated that up to 75% of type 2 diabetic patients may present some form of NAFLD [29]. Moreover, and interestingly, a newly reported finding revealed that NAFLD is now considered a strong determinant for the development of metabolic syndrome (MS), rather than the hepatic manifestation of the MS, as previously emphasized [30]. Great effort is nonetheless being made toward the identification of sensitive diagnostic tests that do not involve invasive procedures to address a common concern in patients with NAFLD—whether they have NASH or simple steatosis. It is essential to permit early detection, timely treatment and its prompt management.

### 4.1. HOMA-IR Score

Given the complexity of the pathogenesis of NASH, it is likely that multiple pathways play a significant role in the development of the disease. Oxidative stress, insulin resistance and systemic inflammation are considered central in the pathogenesis of NAFLD and NASH [31]. Higher HOMA-IR scores are independent clinical predictors for NASH [32]. Our results are in line with these findings, as the highest exponent for prediction of NASH was observed for HOMA-IR (Table 4). These outcomes indicate that one unit increase in HOMA-IR score leads to an average of 1.263-fold increase in the odds of NASH occurrence. Moreover, a recently published multicenter study in Japanese NAFLD patients reported that insulin resistance is correlated with the severity of liver histology [33]. Different HOMA cut-offs were proposed through the literature to define insulin resistance. Similar to Shimada et al. [34] but opposed to Dixon et al. [27], our data suggest that all values equal or higher than 2.5 indicate insulin resistance, hence imply an alarm towards further investigation of steatotic patients in the direction of NASH.

### 4.2. Serum Uric Acid 

In humans, SUA is the final oxidation product of purine metabolism and is excreted in urine. Besides a known fact that hyperuricemia is a cause of gouty arthritis, it has also been linked to the development of hypertension, kidney disease, metabolic syndrome and cardiovascular disease. It has been observed that oxidative stress and lipid peroxidation injury play a major role in the pathogenesis of NAFLD [35]. One of the proposed mechanisms is the induction by hyperuricemia of endothelial dysfunction, insulin resistance, oxidative stress and systemic inflammation [36]. Results from a large observational study involving the US adult population linked elevated uric acid level with increasing severity of NAFLD [37]. Similar findings were reported earlier in a prospective observational study that showed the elevation of SUA levels as an independent predictor of an increased risk for incident NAFLD [38]. Furthermore, hyperuricemia was a common finding in male patients with NAFLD and was independently related to early stage of NASH, as recently noted by Sertoglu et al. [39]. Lately, published findings in non-obese postmenopausal women describe higher SUA levels with a positive and independent association to hepatic steatosis [40]. The newest study by Wu et al. [41] showed that a sex-specific SUA level was independently associated with NAFLD and reported significantly greater SUA levels in females than in males. Our finding of SUA level above 380µmol/L has been noted as an independent predictive factor in NASH detection. Additionally, we detected raised albumin and SH group levels, denoting the significance of hyperuricemia induced oxidative stress in the pathogenesis of NASH. Albumin is the primary source of reduced SH groups that are recognized as potent scavengers of reactive oxygen and nitrogen species [42].

### 4.3. Ferritin

We detected significantly different ferritin levels without iron overload in the NASH group, even though it was still in the referent range when compared to simple steatosis. Sumida et al. [43] included ferritin in their prediction model but suggested higher levels as a cut off than we found in our study. Even more demanding levels were recommended in the previously published research where a threshold serum ferritin >1.5 upper limits of normal was associated with a diagnosis of NASH [44]. Conversely, our findings in regards to ferritin levels in both observed groups are more in line with a study published by Polyzos et al. [23]. However, it has not been clearly established whether the elevated serum ferritin is a consequence of systemic inflammation or a marker of iron overload in patients with NAFLD. Interestingly, even in patients without a hepatic iron overload (such as our patients), a higher serum ferritin was correlated with disease progression. This implies the presence of systemic inflammation that is independent to iron overload [44].

### 4.4. Alanine Aminotransferase

Clinicians need to consider NASH as the most probable cause of unexplained elevation of ALT, usually minor, in a patient with metabolic risk factors. Although ALT is not an ideal biomarker for differentiating simple steatosis from NASH, several studies demonstrated that high levels of ALT are correlated with an increased risk of NASH [45]. Thus far, no optimal ALT level has yet been proposed to predict NASH. Nonetheless, recently published results showed the significantly lower incidence of NASH in normal ALT group compared with a raised ALT group [46]. Our findings appeared to be in line with the findings above. Elevated ALT levels usually initiate further abdominal imaging or histological assessment, which may elucidate why patients with normal ALT levels in our study were older than those with raised ALT levels. This finding is consistent with a study by Fracanzani et al. [47].

### 4.5. Diagnostic Panels

Various diagnostic panels for differentiating NASH from simple steatosis have been previously suggested. In more details, the NAFLD diagnostic panel [48] recommended five markers (diabetes, gender, BMI, triglycerides and cytokeratin-18 (CK18) fragments) and performed better than the NASH diagnostic [49] that included three biomarkers (CK18, adiponectin and resistin) for NASH prediction. The panels showed a good performance with an AUROC of 0.81 and 0.73, respectively. CKs 8 and 18, besides having a structural role in terms of assuring hepatocytes stability, additionally act as a target by toxic stress implying apoptosis/necrosis [50]. In spite of the fact that previous research showed promising results in using CK18 as a useful marker for NASH, more recently published studies cast doubt on its clinical utility. Cusi et al. confirmed the limited value of CK18 as a biomarker of NASH, regarding restrained sensitivity for staging NASH [51]. Again, the study subjects were morbidly obese, so the results may not be adaptable to a patient population with a lower BMI. Moreover, the NAFIC scoring system [43] with a good performance of predicting NASH (AUROC 0.85) used three parameters (ferritin, insulin and type IV collagen 7S), also outperformed several other panels earlier published. The results of this research, nevertheless, may not apply to our patient population as all participants included were Japanese. A statistically significant correlation has been observed with the NAS score and all four parameters included in our HUFA index. On contrary, we did not detect any significant correlation with observed parameters and fibrosis stages. Thus, biochemical parameters used in our index albeit not associated with the fibrosis stage may help early detection of necroinflammation in NAFLD.

The results obtained in this study support the importance of four variables (HOMA-IR, SUA, ferritin and ALT) in differentiating NASH from SS. Hence, their odd ratios indicate a certain increase for one unit raise in parameter values (ranging from 1% to 26%; Table 4) in the odds of the NASH occurrence. Namely, if taken into account the range values of the parameters, the SUA and ferritin for 50 units increase and ALT for 10 units increase may be more clinically relevant. In addition, based on the direct comparison, the performance of our diagnostic index was quite well with an AUROC 0.94. Additionally, we reported a fair sensitivity of 70.3%, excellent specificity of 95.1%, and quite good PPV and NPV (83.1% and 90.0%, respectively). Our model advocates four easy-to-perform, low-cost set of parameters, available in routine clinical practice. The evidence for each of these independently observed parameters supports our hypothesis to use them in a combined prediction model, to distinguish steatosis from early-stage NASH even more accurately, rather than perceive them separately. If all the exclusion criteria are utilized, and the patient fulfills all of the following criteria: HOMA above 2.5, SUA greater than 380 µmol/L, ferritin above 100 µg/L and ALT above 45U/L, NASH may be considered.

The important cost aspect should not be neglected, especially in resource-limited countries such as ours, ensuring parameters/procedures in affordability in the health care system. Furthermore, in a newly published study that provided a cost-utility analysis of a NASH screening [52], liver biopsy confirmation was not found to be cost-effective. In our study more than one third of steatosis patients had undergone a liver biopsy, and steatohepatitis was biopsy confirmed in all participants in the NASH cohort. Since pharmacotherapy for NASH is not clearly established, it is not necessary to perform a liver biopsy in all patients [53]. Furthermore, this invasive modality cannot be suggested for all patients [9]. Thus we performed liver biopsy when clinical data was not conclusive. Strict inclusion criteria and a variety of measured/estimated parameters supported our findings. Nonetheless, our diagnostic index need to be tested and validated in different conditions and cohorts, but can easily be implemented in everyday clinical practice, without additional time and cost.

## 5. Conclusions

We suggest a simple non-invasive HUFA index that encompasses four easily available parameters (HOMA, uric acid, ferritin and ALT) to identify patients with NASH, which may reduce the need for a liver biopsy on a routine basis in patients with NAFLD.

## Figures and Tables

**Table 1:** Anthropometric and clinical data of patient groups.

Characteristic	SS	NASH	*p* value
Male/Female	55/27	18/11	0.001
Age (years)	58 ± 12	49 ± 11	0.527
Weight (kg)	91.7 ± 16.4	96.7 ± 21.3	0.334
Height (m)	176.4 ± 10.3	175.6 ± 9.7	0.775
BMI (kg/m^2^)	29.3 ± 4.7	27.7 ± 10.9	0.883
Waist circumference (cm)	102 ± 11.9	105.9 ± 16.4	0.497
Hip circumference (cm)	106.6 ± 10.2	111.0 ± 15.0	0.794
Waist to hip ratio	0.96 ± 0.02	0.95 ± 0.02	0.965
Systolic BP (mmHg)	125 ± 5	130 ± 5	0.576
Diastolic BP (mmHg)	85 ± 5	85 ± 5	0.988
Pulse (beats/min)	73 ± 3	75 ± 3	0.878

SS: simple steatosis; NASH: nonalcoholic steatohepatitis; BMI: Body mass index; BP: blood pressure.

**Table 2:** Comparative data of parameters in patient groups.

Parameter	SS	NASH	*p* value	Reference Values
ALT, U/L	38.5 ± 17.7	78.2 ± 35.1	0.001	0–41
AST, U/L	26.5 ± 14.3	40.4 ± 18.2	0.001	0–37
AST/ALT ratio	0.7 ± 0.26	0.5 ± 0.16	0.001	-
GGT, median (IQR), U/L	33.5 (21.2–60.5)	32 (20.0–68.5)	0.903	0–55
Glucose, mmol/L	6.0 ± 2.00	6.0 ± 1.04	0.602	3.9–6.1
HbA1c, median (IQR), %	5.4 (5.1–5.7)	5.5 (5.1–5.9) ± 1.3	0.503	3.9–6.1
Total bilirubin, µmol/L	13.7 ± 11.3	15 ± 7.2	0.222	0–20.5
Total proteins, g/L	71.3 ± 14.6	73.4 ± 16.3	0.009	62–81
Albumin, g/L	44.7 ± 9.3	46.6 ± 9.9	0.001	34–55
Sulfhydryl groups, mM	0.50 ± 0.09	0.55 ± 0.07	0.037	0.40–0.50
TC, mmol/L	5.80 ± 1.43	5.20 ± 1.40	0.011	0–5.2
HDL, mmol/L	1.07 ± 0.42	1.01 ± 0.47	0.602	≥1
LDL, mmol/L	3.65 ± 1.35	3.20 ± 1.16	0.039	0–3.4
TG, mmol/L	1.86 ± 1.02	1.95 ± 1.19	0.944	0–1.7
SUA, µmol/L	346.2 ± 84.7	423.5 ± 83.1	0.001	210–460
Urea, mmol/L	5.20 ± 2.02	5.10 ± 1.50	0.815	2.5–7.5
Creatinine, µmol/L	71.70 ± 23.80	75.34 ± 24.60	0.212	59–104
CrCl (M/F), mL/min	111 ± 12.50/95 ± 15.0	106 ± 12.40/95 ± 11.20	0.350	M/F: 97–137/88–128
Ferrum, µmol/L	17.42 ± 6.80	18.91 ± 7.65	0.328	11–30
Ferritin, µg/L	92.38 ± 66.60	135.00 ± 85.42	0.003	20–250
Insulin, pmol/L	99.27 ± 39.00	191.65 ± 110.95	0.001	43–195
Vitamin B12, ng/L	463.60 ± 245.8	486.50 ± 388.6	0.602	211–946
Folate, µg/L	10.45 ± 4.86	12.30 ± 6.29	0.303	3.8–16
Homocysteine, µmol/L	14.80 ± 5.32	15.35 ± 3.30	0.392	5–15
IL-6, pg/mL	3.33 ± 1.50	6.55 ± 14.90	0.890	0.0–7.0
Leptin, ng/mL	14.74 ± 13.93	10.85 ± 12.56	0.157	0.35–28.0
ESR, median (IQR), mm/h	6 (2–8)	10 (6–18)	0.002	M<15/F<20
CRP, median (IQR), mg/L	1.2 (0.5–1.4)	1.7 (0.8–3.6)	0.031	0–3
HOMA-IR	1.94 ± 0.85	3.40 ± 1.06	0.001	<2.5

Data are given as mean ± standard deviation of the mean, otherwise stated in the table. Between-group differences: Mann–Whitney or *t*-test; ALT: Alanine aminotransferase; AST: Aspartate aminotransferase; GGT: Gamma glutamyl transferase; HbA1c: Glycosylated hemoglobin; TC: Total cholesterol; HDL: High-density lipoprotein; LDL: Low-density lipoprotein; TG: Triglycerides; SUA: Serum uric acid; CrCl: Creatinine clearance based on the Cockcroft–Gault equation (adjusted for body weight), M: Male, F: Female; IL-6:Interleukin 6; ESR: Erythrocyte sedimentation rate; CRP: C-reactive protein; HOMA-IR: Homeostatic model assessment of insulin resistance; IQR: Interquartile range.

**Table 3:** Correlation between histological characteristics of patients and proposed parameters in the NASH group.

Histological Finding	NASH *(n* = 29) Median (IQR)	HUFA Index			
		HOMA-IR	SUA	Ferritin	ALT
NAS score (0–8)	4 (4–5)	r_s_ = 0.381*	r_s_= 0.411*	r_s_ = 0.403*	r_s_ = 0.393*
Steatosis (1–3)	2 (2–2)	r_s_ = 0.184	r_s_ = 0.219	r_s_ = 0.061	r_s_ = 0.219
Inflammation (0–3)	1 (1–2)	r_s_ = 0.173	r_s_ = 0.359*	r_s_ = 0.215	r_s_ = 0.353*
Ballooning(0–2)	1 (1–1)	r_s_ = 0.338	r_s_ = 0.345	r_s_ = 0.325	r_s_ = 0.332
Fibrosis (0–4)	1 (1–1)	r_s_ = 0.160	r_s_ = 0.187	r_s_ = 0.177	r_s_ = 0.192

* Correlation is significant at the 0.05 level (two-tailed); r_s_—Spearman’s correlation coefficient; HUFA index is an acronym of the four proposed parameters (HOMA-IR: Homeostatic model assessment of insulin resistance, sUA: Serum uric acid, Ferritin andALT: Alanine aminotransferase). NAS score—NAFLD activity score.

**Table 4:** Combined logistic index for the prediction of NASH.

Variables	B	S.E.	Wald	df	*p* value	Exp(B)	CI (95%)
SUA	0.010	0.005	4.997	1	0.025	1.010	1.001–1.019
Ferritin	0.015	0.006	5.852	1	0.016	1.015	1.003–1.028
ALT	0.023	0.009	6.894	1	0.009	1.023	1.006–1.041
HOMA-IR	1.503	0.388	5.029	1	0.000	1.263	1.001–1.525

CI: Confidence interval.

**Table 5:** Area under the curve for the combined HUFA index.

Area	Std. Error	*p* value	Asymptotic 95% Confidence Interval	
			Lower Bound	Upper Bound
0.940	0.022	0.000	0.897	0.983
